# Supplementary material for: Chromothripsis during telomere crisis is independent of NHEJ, and consistent with a replicative origin
Source: Genome Res. 2019 May;29(5):737–49. doi: 10.1101/gr.240705.118 (PMC6499312; doi:10.1101/gr.240705.118)
Supplement: Supplemental Material [file supp_gr.240705.118_Supplemental_file_1.zip › contigs/annotated_contigs/DB116/contig.2.DB116_length_622_mean_cov_5.78778135048.docx]

**DB116_length_622_mean_cov_5.78778135048**

```
TTTTAGATATAGTAACATTATTGAATATTTGAATGCTTTGCAGTGATGAAATTCTCTCCTATTACTTGAGGCATAGCTAAATACCTGCC
 >chr2:78223360-78223704 + E=3e-195
ATTATGTATACAAATGCATTGACGTCACCCTGTCCTTTCTATCTGTTCTACCTTCAACTTGTCCATTGGATTCACTGCCTAGAGAACAA

CTCAGAGAAACCATACTTCTGCTACTAGCTGCTATGAAAATACCTCTGACATGGAAAGGGTGCAGTTTCTGAATGCCCATATTGCTTCC

AACATTCATGATGCCAGAGCATTGCTGCGTTATAGACATACATGGTAATAACTGCTCCTCCCAAAATCTATA|TGAAA|TGTCAAAATA
                                                                         >chr2:78224465
TTCATATATGTAGAAAATCCAGAATAATTTATAGGCAAACTACTAAAATCAATAGGAAATTATCATTGCTGGGTTCAAGTTTAATATTT
-78224748 + E=1e-158
AGAAAAAATAATTGTAGGCTAGGTGCAGTGGATCACACCTATAATTTCAGCACTTTGGCAGGCCAAAGGTCGGTGGATTGCTTGAGCCC

AGGAGTTCGAGACCAGCCTAGGCAACATATTAGGTTAGTGCAAAAGTAATTGTGGTCTTTGCTATTACTTTAATGGCAAAAAACGCAAC

T
```
